# Commissioning a four‐dimensional Computed Tomography Simulator for minimum target size due to motion in the Anterior–Posterior direction: a procedure and treatment planning recommendations

**DOI:** 10.1002/acm2.12980

**Published:** 2020-07-15

**Authors:** Marcus Sonier, Brandon Vangenderen, Dallas Visagie, Cameron Appeldoorn, Te‐Chih (Archie) Chiang, Lindsay Mathew, Stefan Reinsberg, Jim Rose, Ramani Ramaseshan

**Affiliations:** ^1^ Department of Medical Physics BC Cancer –Abbotsford Centre Abbotsford BC Canada; ^2^ Department of Physics University of British Columbia Vancouver BC Canada

**Keywords:** 4DCT, imaging uncertainty, motion management, reconstruction artifact, SBRT

## Abstract

The purpose of this work is to develop a procedure for commissioning four‐dimensional computed tomography (4DCT) algorithms for minimum target reconstruction size, to quantify the effect of anterior–posterior (AP) motion artifacts on known object reconstruction for periodic and irregular breathing patterns, and to provide treatment planning recommendations for target sizes below a minimum threshold. A mechanical platform enabled AP motion of a rod and lung phantom during 4DCT acquisition. Static, artifact‐free scans of the phantoms were first acquired. AP sinusoidal and patient breathing motion was applied to obtain 4DCT images. 4DCT reconstruction artifacts were assessed by measuring the apparent width and angle of the rod. Comparison of known tumor diameters and volumes between the static image parameters with the 4DCT image sets was used to quantify the extent of AP reconstruction artifact and contour deformation. Examination of the rod width, under sinusoidal motion, found it was best represented during the inhale and exhale phases for all periods and ranges of motion. From the gradient phases, the apparent width of the rod decreased with increasing amplitude and decreasing period. The rod angle appeared larger on the reconstructed images due to the presence of motion artifact. The apparent diameters of the spherical tumors on the gradient phases were larger/equivalent than the true values in the AP/LR direction, respectively, while the exhale phase consistently displayed the spheres at the approximately correct diameter. The Eclipse calculated diameter matched closely with the true diameter on the exhale phase and was found to be larger on the inhale, MIP, and Avg scans. The procedure detailed here may be used during the acceptance and commissioning period of a computed tomography simulator or retroactively when implementing a SBRT program to determine the minimum target size that can be reliably reconstructed.

## 1 INTRODUCTION

Modern treatment of cancers located in the lung or abdomen often involves a motion management technique to ensure the geometric accuracy of a radiotherapy prescription.^1^, ^2^, ^3^, ^4^ One such technique: four‐dimensional computed tomography (4DCT) is based on the premise of obtaining multiple CT scans of a free breathing patient that are correlated with a specific time point in the breathing cycle from an external marker, thus, depicting static snapshots of a patient’s internal anatomy as it changes due to breathing motion.^5^, ^6^, ^7^ Four‐dimensional computed tomography scan data are routinely binned based on one aspect of the recorded breathing cycle: amplitude or phase.^7^, ^8^, ^9^, ^10^ Amplitude binning is accomplished by grouping CT images of identical external marker position in the anterior–posterior (AP) direction which is thought to correlate with amplitude of diaphragm motion.^8^ This method often results in clear images free of blurring artifacts but contains image sets with missing data (CT slices) potentially missing the tumor's location when irregular breathing patterns are present, a typical situation for patients diagnosed with cancer. Phase binning is accomplished by grouping CT images into bins based on segmenting the breathing trace when the images are acquired.^8^ This method often results in blurred images (reconstruction artifact) but contains whole image sets illustrating the entire extent of tumor motion. In External Beam Radiation Therapy (EBRT), it is essential that the full extent of tumor motion be evaluated in order to avoid a geometric miss of the tumor. As a result, phase binning algorithms are typically employed when preparing a patient treatment plan and, in some cases, is the only available method in commercial software.^9^, ^11^

In the case of tumors subject to repetitive motion such as breathing, the entire extent of tumor motion must be treated with the prescription dose to avoid the target moving beyond the boundaries of the treatment area. Four‐dimensional computed tomography provides the necessary anatomic information required for the Radiation Oncologist to delineate the tumor across all breathing phases.^12^ Quality Assurance (QA) of the 4DCT reconstruction algorithm is a necessity to ensure high‐quality patient care. Various guidelines have been published recommending QA procedures for CT and 4DCT software.^13^, ^14^, ^15^ However, these tests are employed strictly in the superior–inferior (SI) direction. While it is true that tumor motion due to breathing primarily occurs in the SI direction, the AP and left–right (LR) motion may also cause reconstruction artifacts deteriorating CT image quality. Multiple publications have reported tumor AP motion to be non‐negligible with some indicating patient‐specific motion in the AP direction as comparable to the SI direction.^15^, ^16^, ^17^ Characterization of 4DCT reconstruction artifacts in the AP direction is therefore underrepresented in the current literature with no recommendations for commissioning procedures to characterize minimum object reconstruction limits.

Stereotactic body radiation therapy (SBRT) is a branch of EBRT that focuses on treating small tumors with a high dose in relatively few treatments where geometric accuracy is of the utmost importance. In the case of lung SBRT, the tumor may not inhibit lung function due to the small size, potentially resulting in a highly mobile target.^18^ Retrospective evaluation of patient treatment plans at our center indicated that reconstruction artifacts manifested more prominently in SBRT patients where small tumor size was standard. Thus, the aim of this work is threefold: (a) to develop a procedure for CT simulator commissioning of 4DCT algorithms to determine minimum reconstruction size of small tumors, (b) to quantify the effect of AP motion artifacts on known object reconstruction for periodic and irregular breathing patterns, and (c) to provide treatment planning recommendations for cases where the target size falls below a minimum threshold.

## 2 MATERIALS AND METHODS

A movable and programmable platform capable of mounting the Dynamic Thorax Phantom Model 008A (CIRS Inc., Norfolk, VA, USA) was constructed by an in‐house machine shop. This platform was designed with a user interface that accepted input to control AP motion in a reproducible and consistent manner. 4DCT images were obtained using a GE Lightspeed RT16 CT scanner (General Electric, Boston, MA, USA) in cine mode (120 kV, 145 mA, 2.5 mm slice thickness, 20 mm collimation) and Real‐Time Position Management (RPM) software (Varian Medical Systems, Palo Alto, CA, USA) with both the rod and thorax phantoms mounted on the movable platform using sinusoidal and irregular breathing trace inputs. Access to patient data to obtain an irregular breathing trace for this study was approved by the institutional ethics committee (Certificate Number H18‐00629).

### 2.A. Platform motion

**Fig. 1:**
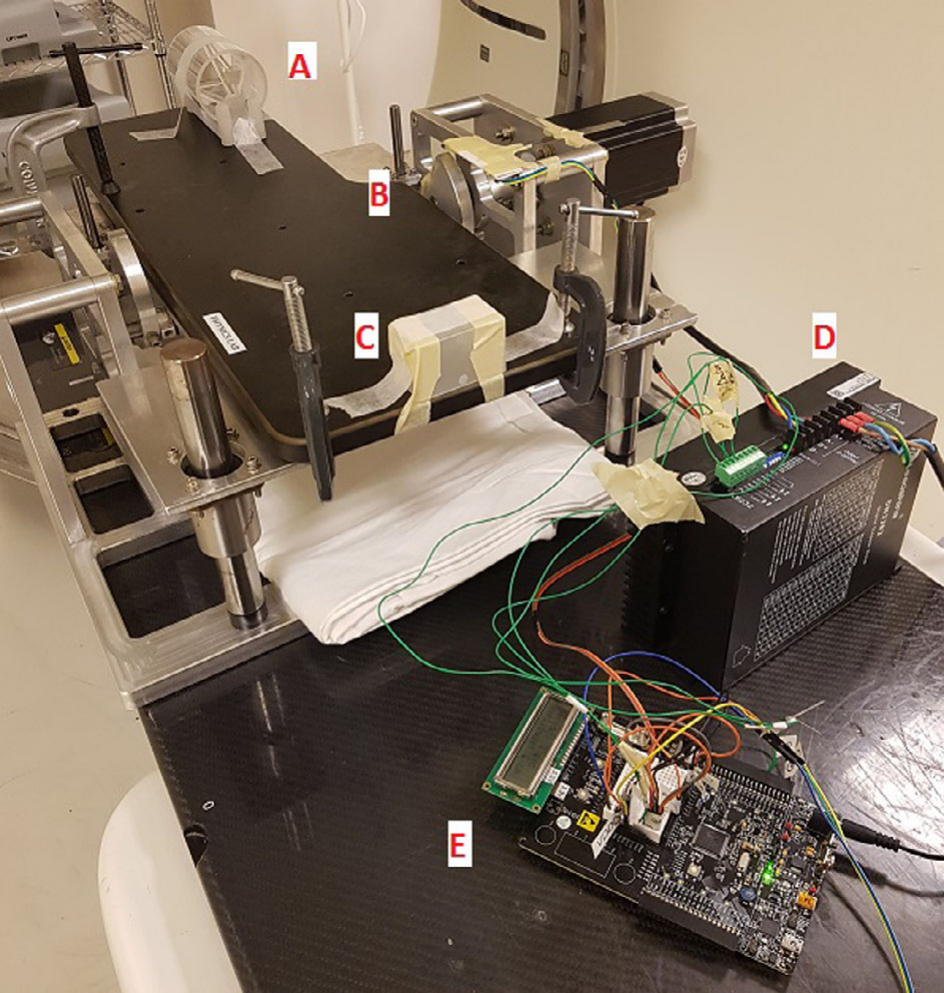
Movable platform with mounted rod phantom. Labels indicate (a) Rod Phantom, (b) rotational motor for platform motion, (c) RPM tracking block, (d) battery power source, and (e) custom programmed microcontroller.

A mechanized platform was used to provide AP motion to the phantoms during 4DCT image acquisition. This device consisted of an aluminum support structure, a carbon fiber board extending beyond the aluminum frame, and a lifting mechanism to raise and lower the board. The lifting mechanism used a high torque stepper motor to rotate an axel connected to a beam that supported the carbon fiber board. As the axel rotated, the support beam traveled in a circular arc changing the AP position of the board at each angle. By mapping the vertical positions of the board to the rotation angle of the axel, the platform could be raised or lowered to match known vertical displacements. Figure 1 shows the platform with the mounted rod phantom.

The platform’s motion was controlled by a Cypress Semiconductor ARM microcontroller (Cypress Semiconductor Corp., San Jose, CA, USA) using in‐house software written in C within the PSoC Creator 4.2 development environment (Cypress Semiconductor Corp., San Jose, CA, USA). Breathing patterns were stored in the form of arrays of numbers which represented the desired vertical displacement of the platform from the home position (middle point). Each position corresponded to a 30 ms step of the breathing pattern. The device was able to move the platform a maximum of ±4 cm vertically from the home position. The software stored patterns as values between 0 and 2 where 0, 1, and 2 were the minimum, home (middle), and maximum vertical positions, respectively. A scaling factor based on the maximum range of motion was used to convert these values to their corresponding physical position in cm.

**Fig. 2:**
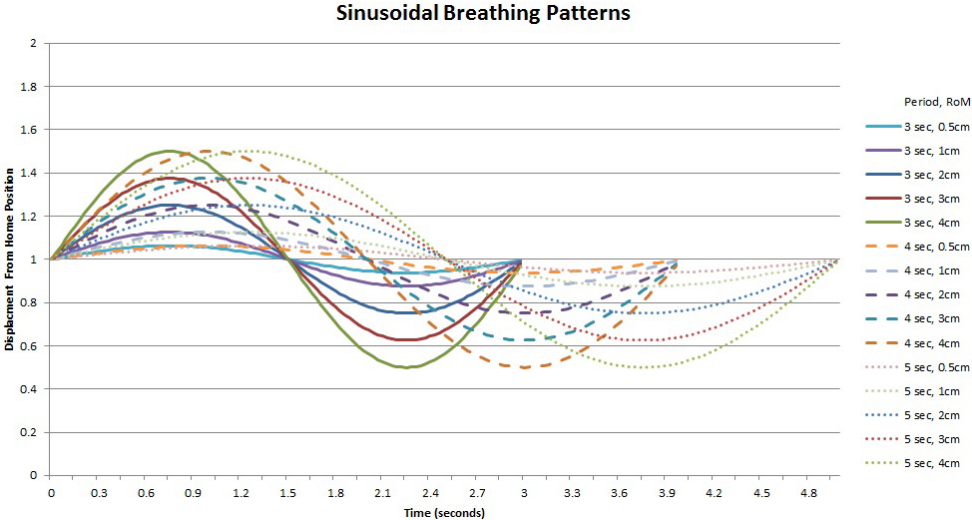
Sinusoidal wave motions applied to the platform during image acquisition for given periods and range of motion (RoM). The minimum, home, and maximum positions of the platform are indicated by values of 0, 1, and 2, respectively.

Microsoft Excel 2010 (Microsoft, Redmond, WA, USA) was used to generate the arrays of values required for the sine wave patterns in 30ms time steps. Each time step was correlated with a point on the sine function to determine the percent of the displacement for that step. Arrays of position values were generated for 3, 4, and 5 s periods with maximum vertical displacements of 0.5, 1, 2, 3, and 4 cm for each period (amplitudes of 0.25, 0.5, 1, 1.5, and 2 cm, respectively). Figure 2 displays the various sine wave motions applied to the platform during image acquisition.

**Fig. 3:**
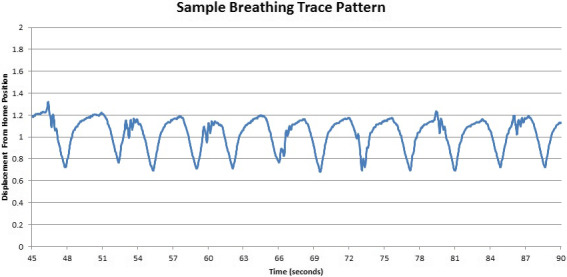
Patient breathing trace sample applied to the platform during image acquisition. The minimum, home, and maximum positions of the platform are indicated by values of 0, 1, and 2, respectively.

Patient breathing data from a sample lung SBRT case were also extracted from a RPM data file and converted into the same form as discussed above (scaled values between 0–2). The maximum vertical position for this breathing pattern was 1.28 cm above the home position and the minimum position was 1.62 cm below the home position which corresponded to a maximum vertical displacement of 2.90 cm. The pattern repeated for 153 s before looping and contained 43 breath cycles with slightly varied periods and occasional bursts of noise caused by stutters, coughs, or other patient breathing behaviors. Figure 3 illustrates a sample of the extracted patient breathing pattern.

### 2.B. Rod phantom and image acquisition

**Fig. 4:**
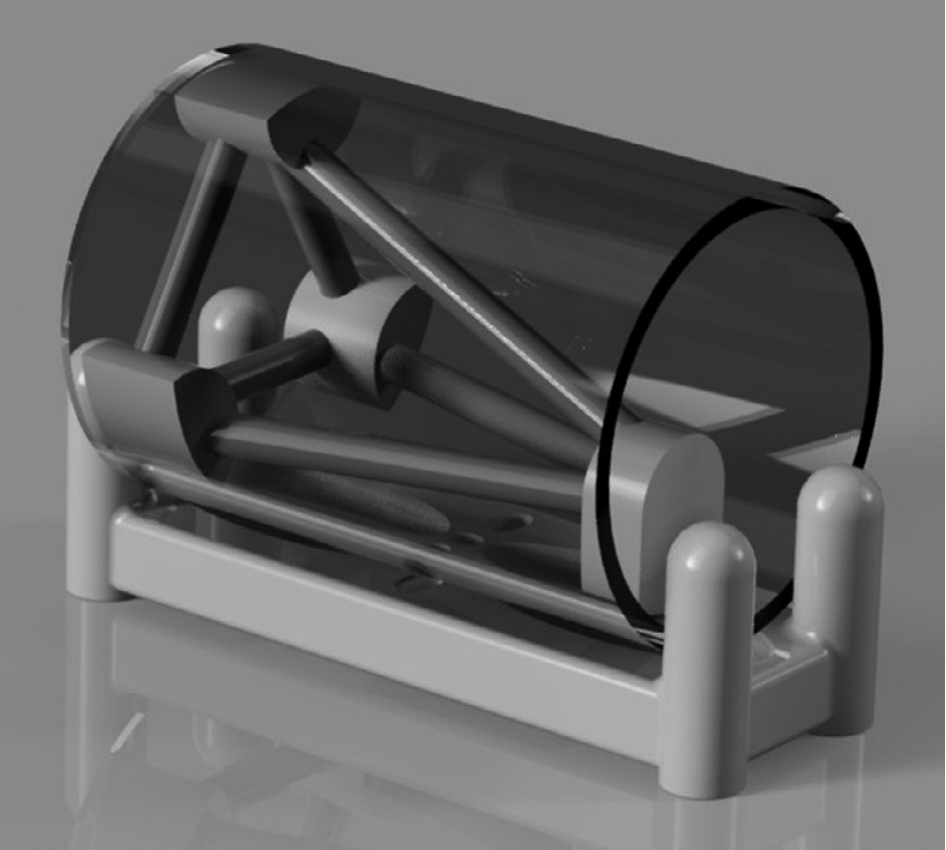
Rendering of the rod phantom.

The rod phantom was constructed in the shape of a pyramid housed within a cylindrical tube as shown in Fig. 4. The primary rod was manufactured to have a diameter of 6.35 mm (¼'') and set at an angle of 26.0° with respect to the CT couch. The phantom was first scanned in a static orientation to acquire a standard CT image set free of motion artifact with a slice thickness resolution of 2 mm. Sinusoidal motion was then applied to the platform to move the rod in the AP direction for each period and maximum displacement as detailed in Fig. 2. The rod phantom was scanned after a few breathing cycles had elapsed when the period measured by the RPM software was within 0.1 s of the applied period. All image sets were acquired using a clinical cine image acquisition protocol, selected for consistent slice width and decreased patient dose as compared to helical scan modes, with the cine time between images and cine duration calculated for each scan using period/10 and period +1, respectively.^19^ One final image set of the rod was obtained by applying the patient breathing pattern to the platform with a cine time between images of 0.4 s and cine duration of 5 s. This same breathing pattern was then applied to the CIRS Dynamic Thorax Phantom Model 008A with a selection of four solid clay spheres with diameters: 1, 2, 3, and 4 cm, inserted into the lung equivalent region in order to determine the effect of the motion artifact on contour deformation when considering a realistic target. All image sets were reconstructed using the Advantage 4D software (General Electric, Boston, MA, USA) via a phase binning algorithm with a 10% bin size and exported to the Eclipse v13.6 Treatment Planning System (Varian Medical Systems, Palo Alto, CA, USA) for analysis.

### 2.C. Data analysis

4DCT reconstruction artifacts were assessed by fixing the window and level to values of 300 and −500, respectively, that displayed the full extent of the rod without saturating the background region determined from the static images. At these settings, the apparent width of the rod and apparent angle formed between the rod and CT couch were measured on the maximum intensity projection (MIP), the average intensity projection (Avg), and at six phases consisting of the inhale (0%), exhale (50%), and four transitional gradient phases (20%, 30%, 70%, and 80%). These measurements were repeated across all 15 sine wave reconstruction image sets and in the analysis of the patient breathing pattern. Comparison of the known tumor diameters and volumes between the manufactured parameters and the static CT image with the 4DCT image sets outlined above was used to quantify the extent of AP reconstruction artifact and contour deformation affecting the artificial tumors.

## 3 RESULTS

**Fig. 5:**
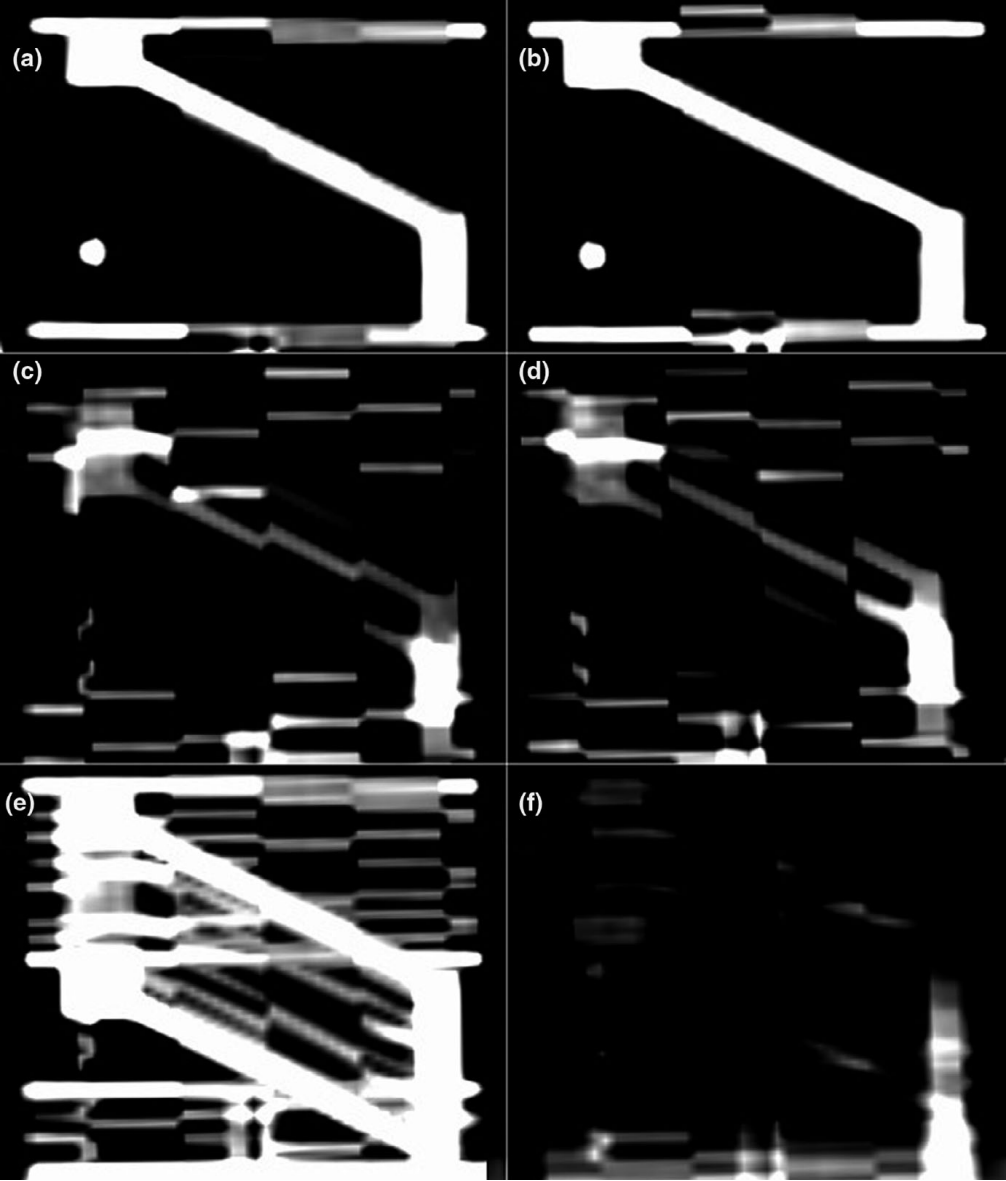
(a) Inhale, (b) exhale, (c) 20% gradient, (d) 80% gradient, (e) MIP, and (f) Avg reconstruction images for a sample 4DCT image set with 5 s period and 4 cm range of motion (2 cm amplitude).

Examination of the rod width, under sinusoidal motion, across each of the six imaging phases found that the diameter was statistically significant and best represented during the inhale (*P* = 0.003) and exhale (*P* = 0.005) phases considering all represented periods and ranges of motion when compared to the gradient phases. Of the remaining four gradient phases, it was noted that the apparent width of the rod decreased with increasing amplitude and decreasing period. Table 1 provides the apparent rod width measured over each imaging phase. Figure 5 shows the inhale, exhale, 20% gradient, 80% gradient, MIP, and Avg reconstruction images for a sample 4DCT image set with 5 s period and 4 cm range of motion (2 cm amplitude). The measured apparent angle of the rod with respect to the CT couch measured larger on the reconstructed images due to the presence of motion artifact. Table 2 provides the apparent rod angle measured over each imaging phase. During sinusoidal motion, the rod angle was statistically significant and most accurately represented on the inhale (*P* = 0.007) and exhale (*P* = 0.004) phases while the apparent angle across the gradient phases was larger than the true angle. Reconstruction phase error (maximum difference between all measured phases and nominal phase) was calculated to be ≤5% for all rod scans as determined by the Advantage 4D software.

**Table 1:** Apparent rod width measured over the MIP, Avg, inhale, and exhale phases along with the mean and standard deviation of the apparent rod width measured over the gradient phases.

Period (s)	Range of motion (cm)	Apparent rod width (cm)				
		MIP	Avg	Inhale	Exhale	Gradient
3	0.5	1.00	0.68	0.64	0.62	0.64 ± 0.01
	1	1.34	0.67	0.62	0.64	0.58 ± 0.02
	2	2.19	0.13	0.64	0.63	0.46 ± 0.01
	3	3.02	0.14	0.70	0.78	0.36 ± 0.02
	4	3.95	0.14	0.58	0.58	0.29 ± 0.05
4	0.5	0.98	0.65	0.62	0.61	0.63 ± 0.01
	1	1.41	0.85	0.62	0.64	0.60 ± 0.02
	2	2.26	0.12	0.65	0.63	0.62 ± 0.14
	3	3.09	0.22	0.63	0.68	0.44 ± 0.02
	4	3.92	0.29	0.63	0.64	0.31 ± 0.02
5	0.5	0.99	0.64	0.64	0.62	0.63 ± 0.01
	1	1.42	0.82	0.62	0.64	0.63 ± 0.02
	2	2.25	0.12	0.64	0.61	0.63 ± 0.07
	3	3.12	0.18	0.63	0.61	0.48 ± 0.04
	4	4.07	0.19	0.64	0.65	0.35 ± 0.04
Breathing trace	2.9	2.45	0.5	0.48	0.64	0.58 ± 0.03

**Table 2:** Apparent rod angle measured over the MIP, Avg, inhale, and exhale phases along with the mean and standard deviation of the apparent rod angle measured over the gradient phases.

Period (s)	Range of motion (cm)	Apparent rod angle (°)				
		MIP	Avg	Inhale	Exhale	Gradient
3	0.5	27.3	27.2	27.7	27.6	27.6 ± 0.2
	1	28.3	27.3	27.5	27.4	27.9 ± 1.2
	2	26.1	28.8	25.9	26.5	27.3 ± 1.0
	3	26.7	27.1	26.9	26.3	27.8 ± 0.3
	4	27.2	29.5	26.4	26.2	27.5 ± 1.1
4	0.5	28.0	28.0	27.9	27.5	27.5 ± 0.4
	1	27.5	27.8	27.6	27.6	27.5 ± 0.7
	2	26.1	27.9	25.9	26.3	26.7 ± 0.5
	3	26.4	31.9	26.2	26.2	27.3 ± 0.6
	4	26.5	27.9	25.2	26.3	27.6 ± 0.9
5	0.5	27.9	27.8	27.6	27.6	27.7 ± 0.6
	1	27.8	27.0	27.7	27.4	27.0 ± 0.4
	2	26.3	29.1	26.1	26.8	27.0 ± 0.2
	3	26.9	27.4	26.0	26.1	26.6 ± 1.0
	4	26.2	28.0	25.7	25.7	27.6 ± 0.4
Breathing trace	2.9	26.8	28.4	25.9	27.4	27.0 ± 0.5

**Fig. 6:**
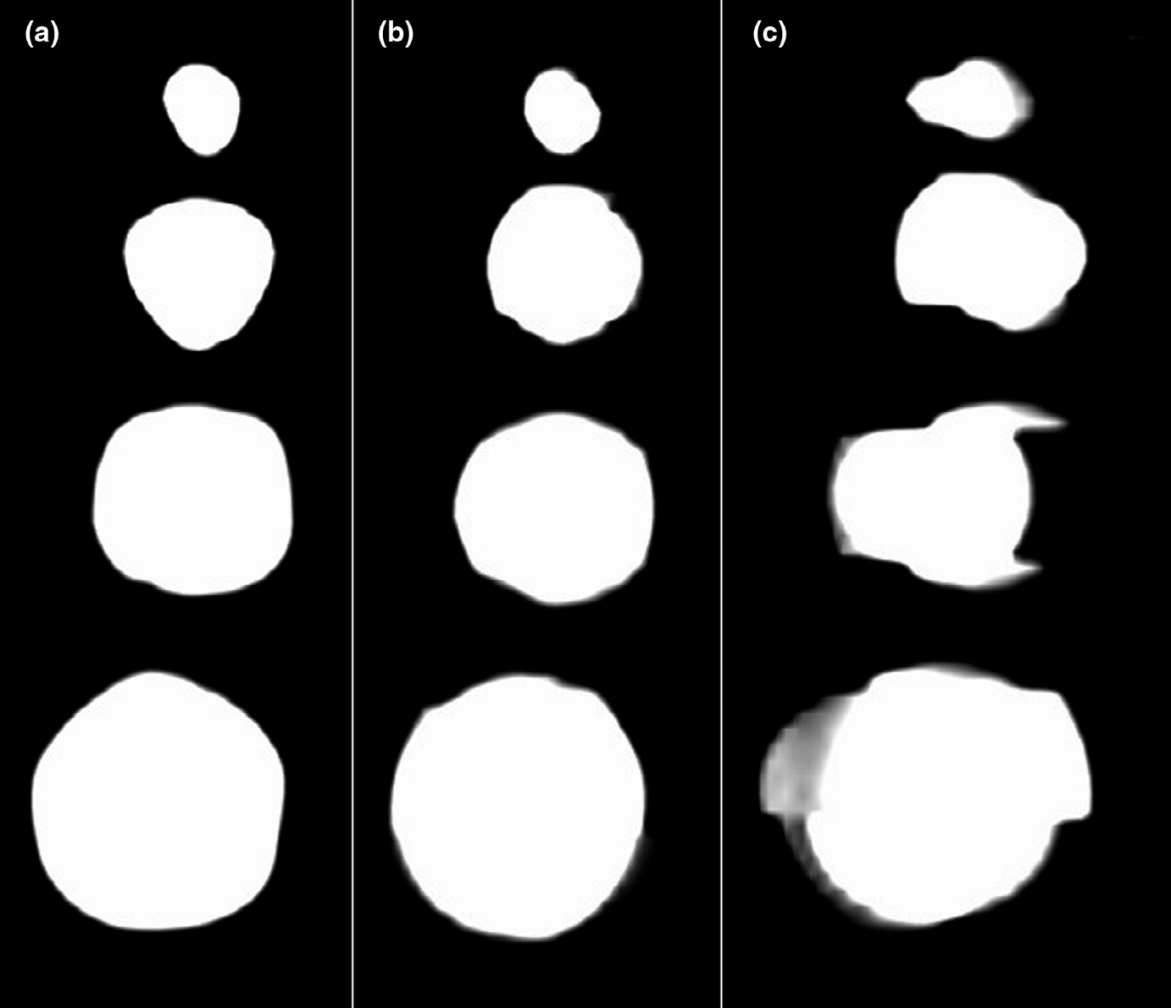
Sample image of each sphere illustrating the apparent expansion of the object due to the reconstruction artifact: (a) static, (b) exhale phase (50%), (c) gradient phase (80%).

The apparent diameters of the spherical clay tumors measured on the gradient phases were found to be larger than the true values in the AP direction and equivalent to the true values in the LR direction while the exhale phase consistently (AP: *P* = 0.05, LR: *P* = 0.11) displayed the spheres at the approximately correct diameter, although not statistically significant. Table 3 provides the measured sphere diameters over each imaging phase. Each sphere was also contoured using an auto‐thresholding Hounsfield unit range of −500 to 2000 on the static image and across all 4DCT reconstruction phases to determine the Eclipse calculated volume and equivalent diameter. Table 4 presents the calculated sphere volumes and equivalent diameters over each imaging phase. The exhale phase most accurately reconstructed each sphere (Volume: *P* = 0.05, Equivalent Diameter: *P* = 0.16) although not statistically significant. Figure 6 shows a sample image of each sphere illustrating the apparent expansion of the object due to the reconstruction artifact. Reconstruction phase error was calculated to be 6% for the spherical tumor scans as determined by the Advantage 4D software.

**Table 3:** Apparent sphere AP and LR diameters measured over the MIP, Avg, inhale, and exhale phases along with the mean and standard deviation of the apparent sphere diameters measured over the gradient phases.

Direction	Apparent sphere diameter (cm)						
	Planned	Static	MIP	Avg	Inhale	Exhale	Gradient
AP	1	1.20	3.03	2.19	1.25	1.27	1.66 ± 0.30
	2	2.36	3.80	3.39	2.92	2.44	2.70 ± 0.19
	3	3.12	4.86	3.54	4.04	3.10	3.41 ± 0.25
	4	3.96	5.66	4.79	4.13	3.94	4.48 ± 0.47
LR	1	1.17	1.27	1.10	1.26	1.18	1.17 ± 0.02
	2	2.10	2.16	2.02	1.98	2.07	2.06 ± 0.03
	3	3.07	3.15	3.03	2.98	3.07	3.07 ± 0.02
	4	3.98	4.01	3.90	3.93	3.90	3.87 ± 0.06

**Table 4:** Calculated sphere volumes and equivalent diameters measured over the MIP, Avg, inhale, and exhale phases along with the mean and standard deviation of the sphere volumes and equivalent diameters measured over the gradient phases.

Calculation element	Sphere diameter (cm)	Image reconstruction					
		Static	MIP	Avg	Inhale	Exhale	Gradient
Volume (cc)	1	1.06	2.65	1.06	1.03	0.94	1.06 ± 0.11
	2	5.90	12.85	8.02	7.61	6.54	7.17 ± 0.45
	3	16.28	25.93	15.33	17.63	15.08	15.38 ± 0.37
	4	34.45	55.58	37.69	37.48	35.25	36.99 ± 1.86
Equivalent diameter (cm)	1	1.3	1.7	1.3	1.3	1.2	1.25 ± 0.06
	2	2.2	2.9	2.5	2.4	2.3	2.43 ± 0.05
	3	3.1	3.7	3.1	3.2	3.1	3.10 ± 0.00
	4	4.0	4.7	4.2	4.2	4.1	4.13 ± 0.05

## 4 DISCUSSION

The inhale and exhale phases consistently reconstructed the rod with apparent widths: 6.33 ± 0.25 mm and 6.39 ± 0.45 mm, respectively, approximate of the true width of 6.35 mm. The gradient phases showed a decreasing apparent rod width with decreasing period (increasing frequency) and increasing amplitude resulting in the highest degree of reconstruction artifact in the 3 s period and 2 cm amplitude (4 cm max range of motion) data set. Examination of the MIP images showed changes in period had no effect on the apparent width of the rod while the apparent width was found to be directly correlated with the amplitude of the phantom motion. The Avg images showed that for a range of motion less than the rod width, the 4DCT image set correctly reconstructed the rod. Comparatively, when the range of motion of the platform was greater than the rod width an increase in motion artifact was present. Notably, when the range of motion exceeded the threshold of 3× the rod width there was a decrease in apparent rod width in the gradient phases resulting in the rod phantom washing out of the images when averaged with the background. The reconstructed rod angle was consistently larger than the true angle due to image blurring present with the inhale and exhale phases closest to the true angle of 26.0° at 26.69 ± 0.90° and 26.77 ± 0.67°, respectively. Consequently, the angle error is not clinically significant in these images since 1° is the standard correction threshold when performing image matching for a patient; however, compounding this error with setup uncertainty at the time of treatment may result in a >1° deviation. In addition, the reconstruction of the rod angle exceeded this 1° threshold on many of the gradient phases, as shown in Table 2, with an added shift of the rod’s center point between adjacent CT slices of up to approximately 25% of the maximum amplitude further illustrating an overall unreliable reconstruction for these image phases when considering increasing AP motion relative to object size. Reconstruction of the rod phantom using the patient breathing trace displayed an increase in image blurring due to the random variations present in the breathing pattern which resulted in larger apparent widths than the sinusoidal breathing trace of comparable range of motion. Notably, the reconstruction of the rod at the exhale phase produced an apparent rod width identical to the true rod width.

Evaluating the diameters of the spherical tumors from the patient breathing trace found that the reconstruction artifacts were more prevalent in the direction of motion (AP) while the LR diameter measurements remained approximately consistent with the static images. Assessment of the gradient phases showed an increase in diameter in the AP direction for all sphere sizes. This result is consistent with the apparent width of the rod in the cases where the amplitude of motion was of comparable size to the true rod width. The exhale phase consistently reconstructed the sphere diameters accurately with a difference of 0.28 ± 0.55 mm while the inhale phase measurements contained more reconstruction error resulting in a difference of 4.25 ± 3.95 mm. Sphere sizes appeared larger on both the MIP and Avg images due to the amplitude of the patient breathing trace being comparable or less than the sphere diameter.

Various studies in the literature have identified that using the MIP reconstruction to contour target structures does not accurately reflect the true target dimensions.^18^, ^20^, ^21^, ^22^ This is especially true in the cases of peripheral tumors that are adjacent to or come into contact with the chest wall which may cover and obscure the tumor in a MIP image due to a higher density. It is therefore recommended to use the gradient phases to account for all possible positions of the tumor across the overall breathing trace. The presence of reconstruction artifacts depicted in this study, however, illustrate that when targets are of a smaller size than the amplitude of breathing motion, the gradient phase images are also unreliable and may result in a significant underestimation of the target size. Notably, the geometric center of the target on each gradient phase tends to remain in the image due to the highest proportion of time spent at that location. As a result, when range of motion exceeds a tumor’s width, this study proposes that the exhale phase be used to characterize a targets shape and size which is then projected onto the geometric center of each gradient phase. The final gross internal target structure may then be defined by a Boolean addition of the target across all imaging phases.

## 5 CONCLUSIONS

The procedure detailed here may be used during the acceptance and commissioning period of a CT simulator or retroactively when implementing a SBRT program to determine the minimum target size that can be reliably reconstructed. For the 4DCT system investigated in this study, the reconstructed object size appears smaller than the true size when the range of motion exceeds the threshold of 3× the object width. In this clinical situation, the exhale phase should be used to characterize a tumor's geometric dimensions for guidance during treatment planning in order to avoid a geometric miss. It is recommended that cancer centers use periodic breathing patterns to characterize the reconstruction limits of their respective 4DCT systems for all combinations of CT simulator and 4DCT software using the commissioning procedure detailed in this study to maximize tumor control probability in small target SBRT cases.

## CONFLICT OF INTEREST

The authors have no conflict of interest to disclose.
